# Comparison of Nutrigenomics Technology Interface Tools for Consumers and Health Professionals: A Sequential Explanatory Mixed Methods Investigation

**DOI:** 10.2196/12580

**Published:** 2019-06-28

**Authors:** Vanessa Araujo Almeida, Paula Littlejohn, Irene Cop, Erin Brown, Rimi Afroze, Karen M Davison

**Affiliations:** 1 University of Hawai'i at Manoa College of Tropical Agriculture & Human Resources Honolulu, HI United States; 2 University of British Columbia Michael Smith Laboratories Vancouver, BC Canada; 3 Kwantlen Polytechnic University Department of Biology Health Science Program Surrey, BC Canada; 4 Fraser Health Authority Clinical Nutrition Abbotsford, BC Canada; 5 Vancouver General Hospital Clinical Nutrition Vancouver, BC Canada; 6 University of Washington School of Public Health Seattle, WA United States; 7 Neighborhood House Washington Tukwila, WA United States; 8 University of Hawai'i at Manoa College of Social Sciences Honolulu, HI United States

**Keywords:** nutrigenomics, nutrigenetics, genomics, epigenomics, interface, user-computer

## Abstract

**Background:**

Nutrigenomics forms the basis of personalized nutrition by customizing an individual’s dietary plan based on the integration of life stage, current health status, and genome information. Some common genes that are included in nutrition-based multigene test panels include CYP1A2 (rate of caffeine break down), MTHFR (folate usage), NOS3 (risk of elevated triglyceride levels related to omega-3 fat intake), and ACE (blood pressure response in related to sodium intake). The complexity of gene test–based personalized nutrition presents barriers to its implementation.

**Objective:**

This study aimed to compare a self-driven approach to gene test–based nutrition education versus an integrated practitioner-facilitated method to help develop improved interface tools for personalized nutrition practice.

**Methods:**

A sequential, explanatory mixed methods investigation of 55 healthy adults (35 to 55 years) was conducted that included (1) a 9-week randomized controlled trial where participants were randomized to receive a standard nutrition-based gene test report (control; n=19) or a practitioner-facilitated personalized nutrition intervention (intervention; n=36) and (2) an interpretative thematic analysis of focus group interview data. Outcome measures included differences in the diet quality score (Healthy Eating Index–Canadian [HEI-C]; proportion [%] of calories from total fat, saturated fat, and sugar; omega 3 fatty acid intake [grams]; sodium intake [milligrams]); as well as health-related quality of life (HRQoL) scale score.

**Results:**

Of the 55 (55/58 enrolled, 95%) participants who completed the study, most were aged between 40 and 51 years (n=37, 67%), were female (n=41, 75%), and earned a high household income (n=32, 58%). Compared with baseline measures, group differences were found for the percentage of calories from total fat (mean difference [MD]=−5.1%; Wilks lambda (λ)=0.817, *F*_1,53_=11.68; *P*=.001; eta-squared [η²]=0.183) and saturated fat (MD=−1.7%; λ=0.816; *F*_1,53_=11.71; *P*=.001; η²=0.18) as well as HRQoL scores (MD=8.1 points; λ=0.914; *F*_1,53_=4.92; *P*=.03; η²=0.086) compared with week 9 postintervention measures. Interactions of time-by-group assignment were found for sodium intakes (λ=0.846; *F*_1,53_=9.47; *P*=.003; η²=0.15) and HEI-C scores (λ=0.660; *F*_1,53_=27.43; *P*<.001; η²=0.35). An analysis of phenotypic and genotypic information by group assignment found improved total fat (MD=−5%; λ=0.815; *F*_1,51_=11.36; *P*=.001; η²=0.19) and saturated fat (MD=−1.3%; λ=0.822; *F*_1,51_=10.86; *P*=.002; η²=0.18) intakes. Time-by-group interactions were found for sodium (λ=0.844; *F*_3,51_=3.09; *P*=.04; η²=0.16); a post hoc analysis showed pre/post differences for those in the intervention group that did (preintervention mean 3611 mg, 95% CI 3039-4182; postintervention mean 2135 mg, 95% CI 1564-2705) and did not have the gene risk variant (preintervention mean 3722 mg, 95% CI 2949-4496; postintervention mean 2071 mg, 95% CI 1299-2843). Pre- and postdifferences related to the *Dietary Reference Intakes* showed increases in the proportion of intervention participants within the acceptable macronutrient distribution ranges for fat (pre/post mean difference=41.2%; *P*=.02). Analysis of textual data revealed 3 categories of feedback: (1) *translation of nutrition-related gene test information to action*; (2) *facilitation of eating behavior change,* particularly for the macronutrients and sodium; and (3) *directives for future personalized nutrition practice*.

**Conclusions:**

Although improvements were observed in both groups, healthy adults appear to derive more health benefits from practitioner-led personalized nutrition interventions. Further work is needed to better facilitate positive changes in micronutrient intakes.

**Trial Registration:**

ClinicalTrials.gov NCT03310814; http://clinicaltrials.gov/ct2/show/NCT03310814

**International Registered Report Identifier (IRRID):**

RR2-10.2196/resprot.9846

## Introduction

### Background

Since the success of the Human Genome project, scientific technology has advanced rapidly in several disciplines, including medicine and nutrition. Given that the interaction of nutrients with DNA can impact nutritional status and the development of complex diseases, nutritional genomics (nutrigenomics) has become increasingly important in nutrition practice. Nutrigenomics encompasses nutrigenetics, which investigates the effect of genetic variation on nutrient bioavailability and metabolism, and nutrigenomics, which examines how nutrients and bioactive food compounds affect human health through epigenetic modifications [1-5]. Furthermore, it forms the basis of personalized nutrition by customizing an individual’s dietary plan based on the integration of life stage, current health status, and genome information. Some common genes that are included in nutrition-based multigene test panels include *CYP1A2* (rate of caffeine break down) [6,7], *MTHFR* (folate usage) [8-10], *NOS3* (risk of elevated triglyceride levels related to omega-3 fat intake) [11], and *ACE* (blood pressure response in related to sodium intake) [12].

Although the advancement of nutrigenomics-based personalized nutrition shows significant promise in improving population health, it also presents challenges. These issues include concerns about the complexity in translating gene-based results into meaningful recommendations that will lead to positive health outcomes [13-15]. Despite these issues, studies have shown that those who receive personalized nutrition interventions based on gene test results show improvements in the quality of their diet [16-19]. However, differences in dietary intakes are not always observed between *risk* and *nonrisk* groups [20], and dietary changes have not been consistently observed across all identified risk gene variants (eg, *MTHFR*) where nutrition advice is provided [16-19]. For nutrigenomics and personalized nutrition to advance in health practice, better interface educational tools (eg, Web applications and targeted messaging after personalized nutrition advice provided) need to be developed that are easily implemented by practitioners, that are understood by consumers, and that foster positive eating behavior changes. Furthermore, they need to incorporate accepted nutrition guidelines, integrate phenotypic information about current health status, and align with behavior change theory principles [5,20]. This study proposes to compare standard and tailored personalized nutrition approaches based on gene testing and to elicit participant feedback about their experiences with the 2 types of interventions. The study results are intended to generate data that will improve nutrigenomics-based education tools for consumers and health professionals.

### Objectives

The main study objectives were to compare a practitioner-facilitated personalized dietary approach that uses genotypic and phenotypic information with a self-driven approach and their impacts on changing participant’s knowledge, motivation, and behavior related to eating habits, the quality of their diet, and the quality of their life. It was hypothesized that significantly higher levels of knowledge, motivation, behavior, and quality of life would be reported and that there would be a higher level of diet quality changes in the group that receives personal DNA diet information plus customized dietary advice (practitioner led) compared with the group that is provided personal DNA diet information (direct-to-consumer, self-driven approach) only. In addition, self-efficacy was evaluated as a potential mediator/moderator of dietary changes and outcomes. Focus group interview data were also collected to facilitate interpretation of the intervention data.

## Methods

### Study Design

The sequential explanatory mixed methods investigation consisted of (1) a randomized controlled trial (2:1 allocation ratio) comparing standard self-driven versus practitioner-facilitated approaches that use DNA-based diet information and (2) qualitative investigation of participants’ experiences to help interpret the intervention’s quantitative outcomes. The study protocol, including paper-based or Web-based data collection forms, was approved by Quorum Institutional Review Board (protocol #32220CDN/1). All the participants provided initial Web-based consent to collect eligibility screening information and, if eligible, baseline information related to their health. At the participant’s first site visit, a second written consent form was reviewed, which participants signed to confirm their continued involvement in the study. The protocol was registered with the US National Library of Medicine (trial registration #NCT03310814) and is also detailed elsewhere [21].

#### Participants and Setting

The participants selected for the study included healthy medically stable adults (aged 35 to 55 years) residing in the greater Vancouver area of the province of British Columbia, Canada. The participants were recruited via social media, a newspaper article, and posters. The eligibility criteria included that they wanted to improve their health, could understand and provide informed consent, and were willing to provide a buccal swab for DNA testing. The exclusion criteria are specified in Multimedia Appendix 1.

#### Description of Study Groups

The participants were randomized using a random number generator by a statistician independent to the study into either the intervention or control (C) group. The intervention (I) group received their gene test result report (standard) plus a personalized nutrition plan that integrated information about their gene test results, health information, personal goals, and dietary intakes (based on nutrient analysis of 3-day food records collected at baseline). Their DNA report results and personalized nutrition plan were reviewed with a trained research registered dietitian (RD) who counseled them about the DNA-based diet recommendations derived from information about their gene markers and variants. For example, if a person possessed a dietary fat utilization–based genotype that was associated with increased risk of a health outcome (eg, dyslipidemia), they were provided *targeted* directives about types (eg, omega-3 fatty acids) and amounts of fat intake (eg, try to eat less than 80 g of fat per day). If a participant did not possess the specified dietary fat utilization–based risk variant, they received the standard *Dietary Reference Intakes* –based recommendations [22,23]. Where possible, recommendations based on the gene test results were clustered to provide manageable and meaningful dietary guidance to the participant (Table 1). As the final step in nutrition consultation, the RD worked collaboratively with the participant to define up to 3 targeted nutrition-related goals that were entered into the Web-based nutrition assessment questionnaire and written in the personalized nutrition report. Both groups received 3 follow-up emails (1 every 2 weeks post intervention) with information about gene-based personalized nutrition as well as tips and reminders (eg, information about food label reading) to help them reach their nutritional goals. After the study’s completion, participants in the C group had the option to have dietary counseling from the research RD.

**Table 1:** Description of gene test.

Component measured	Nutrient or food subcomponent tested^a^
Diet management	Carbohydrates^b^; cholesterol—high density lipoprotein and low density lipoprotein^c^; fat—dietary, stored, monounsaturated, and saturated^b^; insulin sensitivity; and protein^d^
Weight response	Body mass index
Food tolerances	Alcohol; caffeine; gluten; lactose; salt^e^; and sugar craving^f^
Food taste and preferences	Caffeine preference; carbohydrate^b^ preference; fat preference^c^; protein preference^d^; and taste: bitter, salt^e^, and sweet^f^
Vitamins, minerals, and essential fats	Vitamins A, B_6_, B_9_ (folate), B_12_, C, D^g^, and E; calcium^g^; iodine; iron; and omega 3 and 6^c^

^a^Dietary advice was clustered by subcomponents labeled with the superscripted letters b to g.

^b^Carbohydrate-related advice.

^c^Fat-related advice.

^d^Protein-related advice.

^e^Salt-related advice.

^f^Sugar-related advice.

^g^Vitamin D and calcium-related advice.

#### Study Visits

The 4-month study consisted of 6 stages.

##### Recruiting/Screening

This included the initial eligibility screen and, where applicable, baseline assessment (Web based) that collected information about sociodemographics; current health status (eg, presence of health conditions and medication and supplement usage); quality of life; self-efficacy; questions about knowledge, motivation, and action related to DNA-based information; stage of change; physical and sedentary activities; food intakes (food frequency and food selection); anthropometrics; and sleep quality. In addition, participants were sent 3-day food records to complete within 7 (±3 days) of their first site visit.

##### Baseline Assessment

A site visit was conducted with the RD who reviewed the participant’s baseline health assessment information and food records; collected a buccal cheek swab sample; and measured the participant’s height, weight, and waist and hip circumference according to standardized protocols [24].

Bar-coded buccal DNA samples were collected using Oracollect-DNA OCR-100 swabs (DNA Genotek) and stored between 15° and 30°C. Participants did not eat or drink at least 30 minutes before buccal sample collection. The samples were analyzed using Agena MassArray at the Clinical Genomics Centre at Mount Sinai Hospital. The gene tests included 5 evidence-based components (Table 1) [25].

##### Personalized Nutrition Visit or Standard Report Distributed

**Figure 1:**
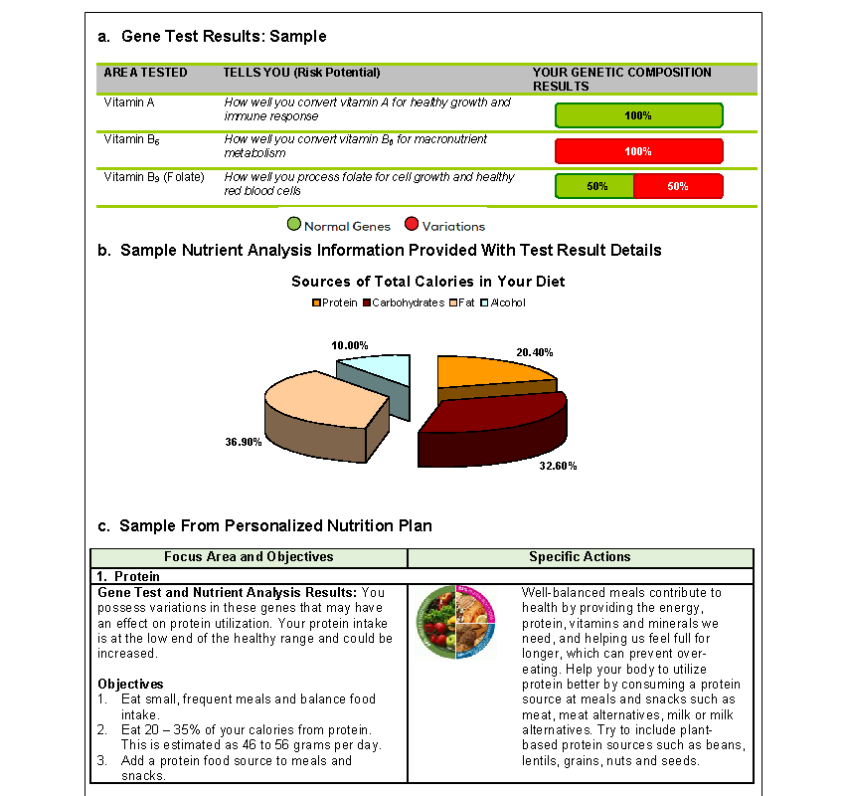
Examples from educational tools.

I group participants came to the site to have their personalized nutrition report reviewed (see Figure 1 for samples from the interface tools); those in the C group received an email with the standard report.

##### Follow-Up #1

A Web-based survey was sent to I and C participants at week 3 post intervention to collect data about any changes in income; social support; and knowledge, behavior, and action; stage of change; and adverse event information. For the I group, questions that asked about whether knowing one’s personal DNA helped with eating behavior change were also included.

##### Follow-Up #2

At 6 weeks after the intervention, the participants received the Web-based baseline health assessment questionnaire and food records to complete in preparation for the final onsite visit (week 8 post intervention). Additional questions were asked about whether having the diet-based DNA information led to action in areas such as label reading and making healthier food choices when eating out, at the grocery store, and at home.

##### Final Visit

The final site visit (I and C groups) included a review of the participant’s health assessment and food record information. Anthropometric measures were repeated. The C-group participants received their individualized gene test–based diet plans and had the option to book appointments with the RD to have them reviewed. Focus groups were then conducted to solicit feedback about participants’ responses to their gene test results and RD consultation as well as about barriers, facilitators, and targets for improvement related to nutrigenomics-related education.

#### Data Collection Tools and Outcome Measurements

FluidSurveys software [26] was used to construct the Web-based closed questionnaires. The Web-based questionnaires were developed using the Checklist for Reporting Results of Internet E-Surveys [27], standard measurements (detailed in measurements section), and protocols for nutrition assessment [25]. All questionnaires contained 12 or less screens (pages) and were pilot tested with study staff and student volunteers (n=11) to assess the usability and technical functionality. The participants were emailed instructions and the links to each Web-based questionnaire at the appropriate times during the study. Quality control data collection procedures included ensuring all questions in each questionnaire were completed, following up with participants about responses where required, and deleting any duplicate entries according to internet protocol addresses. All the data were collected and stored in accordance with Quorum institutional review board guidelines. Copies of the questionnaires may be obtained by emailing the corresponding author. The outcome measures are described in the following sections.

#### Outcomes

##### Nutrition-Related Outcomes

All dietary intake data collection was done according to standard procedures [24,28]. The 3-day food records measured pre/post caloric, macronutrient, micronutrient, and food group intakes, which were compared with nutrition standards (eg, *Eating Well with Canada’s Food Guide* and *Dietary Reference Intakes*). The Canadian version of the Healthy Eating Index (HEI-C) [29] was used to assess diet quality. The Web-based questionnaires also included food selection questions about the types of food consumed, dietary restraint, food insecurity, motivation to change diet, and eating behavior changes. These were based on validated measures [30,31] and current literature related to measuring motivation and eating behavior changes.

##### Health-Related Quality of Life Short Form 8

The Health-Related Quality of Life Short Form 8 is a validated measurement tool of quality of life, functional health, and well-being [32] based on a 4-week recall period. Each item has a 5- or 6-point response range. Higher summary physical and mental component scores indicate better health [33].

##### General Self-Efficacy

General Self-Efficacy (GSE) is a validated item measure of self-efficacy shown to correlate with emotion, optimism, and work satisfaction [34]. It contains 10 questions each with 4 categories of responses that include not at all true, hardly true, moderately true, and exactly true. The scores for each question range from 1 to 4 and the total score is calculated by finding the sum of all items. The scale score ranges between 10 and 40; higher scores indicate greater self-efficacy. The GSE has high reliability, stability, and construct validity that have been confirmed (Cronbach alpha ranges from .86 to .94) [35].

##### Measures of Change in Knowledge, Motivation, and Behavior

Overall, 3 questions to assess for changes in knowledge, motivation, and behavior related to DNA-based dietary advice were developed by the authors based on the Stages of Change Model [36] and current review of the evidence. They were pilot tested among 11 young adults (aged 20 to 40 years) to assess for comprehension and face validity.

### Covariates

The covariates related to dietary intake and health behavior that were measured included the following.

#### Natural Health Products Usage

Data about natural health products (NHP; eg, micronutrients and botanicals) included type (natural product number), dose, and frequency of use. Participants who were taking NHPs were advised at baseline to keep the type, dose, and frequency the same throughout the study.

#### Physical and Sedentary Activities

The physical activity index [37] collected data about the frequency, duration, and intensity of participation in certain activities in the previous 3 months to derive a score that represented the average daily energy expended on leisure time physical activity. A composite measure of sedentary activities was based on total screen time in the previous 7 days [38].

#### Sleep Quality

The Patient-Reported Outcomes Measurement Information System Sleep Disturbance scale-short form [39] was used to assess sleep quality over the previous 7 days. Individual scale items are scored from 1 to 5 and the total scores range between 8 and 40. Lower scores represent a lesser degree of sleep-related impairments.

#### Stress

Measures of stress were based on 2 validated questions from the Canadian Community Health Survey [40].

#### Anthropometrics

Body mass index (kg/m^2^) and waist-to-hip ratio were calculated from standardized height, weight, waist circumference, and hip circumference measures [24].

#### Sociodemographics

Measures of sex/gender, age, relationship status, income, race/ethnicity, and perceived social support were included.

#### Adverse Events

At each study contact point, the participants were asked if they experienced any adverse events or change in and/or had started any new medications or NHPs.

### Data Analysis

#### Quantitative Analysis

##### Food Intake and Nutrient Analysis

Nutrient analysis was conducted using ESHA—The Food Processor Nutrition Analysis and Fitness software and the Canadian Nutrient File [41,42]. Averages of the 3 days of nutrient values were used in the analysis. The food frequency questionnaire (FFQ) values were used to derive usual intakes of the nutrients of interest [24].

##### Descriptive and Inferential Analysis

Means (SDs) or medians (and interquartile range) were reported based on a given continuous variable’s distribution. Subject characteristics, group comparisons, and pre- and postintervention differences were analyzed using the Student *t* tests, binomial tests of 2 proportions, Fisher exact tests, and 2-way repeated-measures analysis of variance with Bonferroni post hoc tests, where appropriate. All analyses were done on an intent-to-treat basis using STATA software [43].

#### Qualitative Analysis

Textual data from the Web-based questionnaires (eg, participant’s personal dietary goals) were grouped into categories where feasible. Data from the focus groups were transcribed, entered into NVivo [44], and analyzed by research team members using interpretative thematic analysis to identify patterns, concepts, themes, and examples in relation to existing behavior change theories and the study objectives [45]. Interpretations were reviewed by research team members and participants to check for descriptive and interpretative validity. Qualitative data were reported based on thematic analysis derived from 3 independent reviews of the textual data.

## Results

### Sample

**Figure 2:**
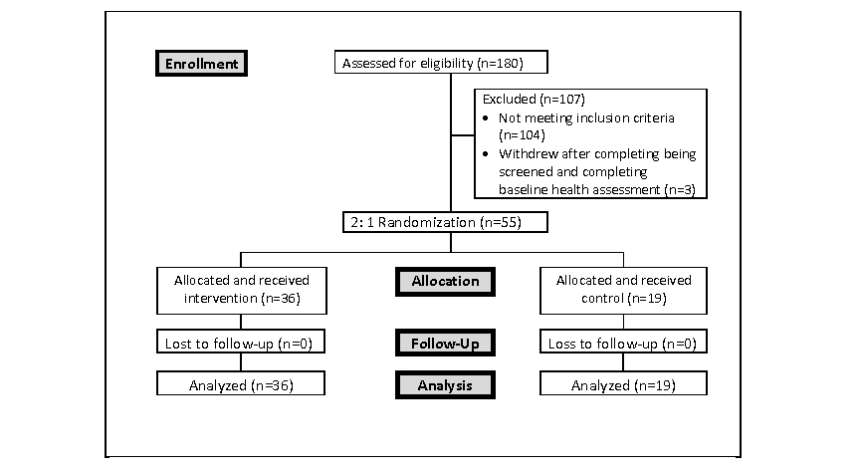
Participant selection.

A total of 478 persons expressed interest in study participation. These individuals were invited sequentially from the recruitment list and balanced by sex/gender, where possible. However, the ratio of females to males who were interested in the study was approximately 8:1, and although all males on the recruitment list were contacted, there was an imbalance in the male/female participant ratio. Of the 478 individuals, a total of 180 (38%) were invited to complete the Web-based eligibility screening questionnaire (Figure 2); 73 of the invited individuals (73/180, 41%) were deemed eligible. Of the 73 eligible individuals, 58 enrolled and 55 (95%) completed the baseline health assessment questionnaire and food records (55/73, 75%). The final sample consisted of 55 adults aged between 37 and 57 years (mean 45.8 years, SD 5.8). Most participants were women (n=41, 74.6%), had graduated from a university or college (n=34, 62%), earned an income higher than Can $90,000 (n=32, 58.8%), and were in a relationship (n=47, 86%). Almost 51% (n=28) of the participants believed they were eating a healthy diet, about two-thirds indicated their health status was *good* (n=25, 46%) or *very good* (n=14, 26%), and 91% (n=50) reported they had favorable social support networks. There were no significant differences between the I and C groups based on various baseline characteristics (Table 2). In addition, the proportion of participants with the gene risk variants did not differ between the I and C groups (proportion range 6% to 69%; *P* values range from .27 to .77).

**Table 2:** Description of sample—baseline characteristics.

Demographics and health-related factors	Intervention (n=36)	Control (n=19)	*P* value
Age (years), mean (SD)	45.4 (6.3)	46.6 (5.0)	.49
In a relationship, n (%)	29 (81)	18 (95)	.47
Postsecondary education, n (%)	20 (56)	14 (74)	.35
Income above Can $90,000, n (%)	19 (53)	13 (68)	.13
Inactive, n (%)	20 (56)	14 (74)	.35
General Self-Efficacy, mean (SD)	32.8 (4.0)	32.7 (4.4)	.88
Health-related quality of life, mean (SD)	62.6 (12.0)	64.6 (9.5)	.53
Body mass index, mean (SD)	28.7 (5.5)	28.1 (4.2)	.69

### Changes in Knowledge, Attitudes, and Behavior

At baseline, more than 90% of respondents (minimum 51) indicated that the diet-related gene test information helped them to understand their health better as well as motivated them to take action related to healthy eating (Multimedia Appendix 2). The proportions of individuals who agreed to statements about taking action on reading nutrition labels more often, selecting healthier food choices at restaurants, purchasing healthier food, making healthier meals at home, taking supplements to support their DNA, and adjusting their eating according to their DNA information were high across both groups; consistently higher proportions were found in the I group. Responses to Stages of Change questions indicated that those in the I group were more likely to report the intention of making healthy changes to their diets within the next month (Multimedia Appendix 2). Responses to questions given to the I group about diet-related changes that were asked at 3 weeks postintervention indicated that 31% (n=11) starting to take supplements, 81% (n=29) took diet-specific actions, and 42% (n=15) indicated they adjusted their eating according to their DNA information.

### Changes in Dietary Intake, Anthropometrics, Self-Efficacy and Quality of Life

**Figure 3:**
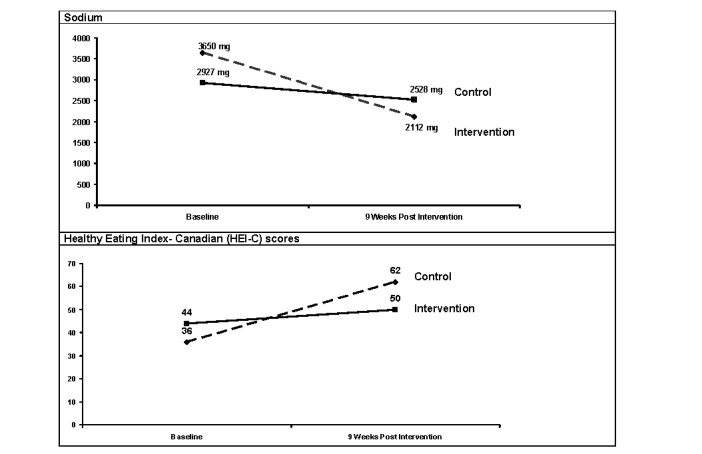
Time-by-group interactions for sodium and diet quality scores.

Significant pre-/postintervention differences (Table 3) were found for the percentage of calories from total fat (mean difference [MD]=−5.1%; Wilks’ lambda [λ]=0.817; *F*_1,53=_ 11.68; *P*=.001; eta-squared [η²]=0.183 ) as well as from saturated fat (MD=−1.7%; λ=0.816; *F*_1,53_=11.71; *P*=.001; η²=0.18) and health-related quality of life (HRQoL) scores (MD=8.1 points; λ=0.914; *F*_1,53_=4.92; *P*=.03; η²=0.086). There were significant differences between groups over time for sodium (λ=0.846; *F*_1,53_=9.47; *P*=.003; η²=0.15) and HEI-C scores (λ=0.660; *F*_1,53_=27.43; *P*<.001; η²=0.35; Figure 3).

When group assignment was stratified by phenotypic plus genotypic information, improved total fat (MD=−5%; λ=0.815; *F*_1,51_=11.36; *P*=.001; η²=0.19) and saturated fat (MD=−1.3%; λ=0.822; *F*_1,51_=10.86; *P*=.002; η²=0.18) intakes were indicated. In addition, significant differences between groups over time for sodium (λ=0.844; *F*_3,51_=3.09; *P*=.04; η²=0.16) with post hoc analysis showing pre/post differences for those in the I group that did have the risk variant (premean 3611 g, 95% CI 3039-4182; postmean 2135 g, 95% CI 1564-2705) and did not have the risk variant (premean 3722 g, 95% CI 2949-4496; postmean 2071 g, 95% CI 1299-2843). Improvements in omega 3 fatty acid intakes were close to significant (MD=0.34 g; λ=0.926; *F*_1,51_=3.99; *P*=.051; η²=0.07). Pre/post differences related to *Dietary Reference Intakes* showed increases in the proportion of intervention participants within the acceptable macronutrient distribution ranges for fat (pre/post difference=41.2%; *P*=.02).

For self-efficacy and quality-of-life measures, there were significant group pre/post intervention differences for HRQoL scores only (MD=8.1 points; λ=0.914; *F*_1,53_=4.92; *P*=.03; η²=0.086).

**Table 3:** Pre- and postintervention comparisons of nutrient intakes, overall diet quality, Health-related Quality of Life Scores, and General Self-Efficacy scores.

Nutrient (units)		Mean difference		Wilks’ lambda (λ)	*F* (df)^a^	eta-squared (η²)	*P* value
		Intervention	Control				
**Macronutrients and food components**							
	Fat (% total calories)	−5.1	−2.9	0.817	11.68 (1,53)	0.183	.001
	Saturated fat (% total calories)	−1.3	1.6	0.816	11.71 (1,53)	0.184	.001
	Omega 3 (g^b^)	−0.4	0.4	1.000	0.02 (1,53)	0.000	.88
	Omega 3 (% total calories)	0.1	0.3	0.964	1.93 (1,53)	0.036	.17
	Omega 6 (g)	−2.2	−3.4	0.688	23.58 (1,53)	0.312	<.001
	Omega 6 (% total calories)	0.8	0.6	0.908	5.26 (1,53)	0.092	.03
	Sodium (mg^c^)	−1537.9	−398.6	0.846	9.47 (1,53)	0.150	.003^d^
	Sugar (g)	1.8	−0.1	0.979	1.11 (1,53)	0.021	.30
	Fiber (g)	−1.2	−1.2	0.000	9.47 (1,53)	0.000	.99
**Vitamins and minerals**							
	Vitamin B_6_ (mg)	−1.6	0.4	0.989	0.58 (1,53)	0.011	.45
	Vitamin B_9_ (mcg DFE^e^)	−89.1	−57.0	0.950	2.72 (1,53)	0.050	.11
	Vitamin B_12_ (mcg^f^)	-0.4	−1.7	0.883	6.90 (1,53)	0.117	.011
	Vitamin D (IU^g^)	1.2	2.2	1.000	0.01 (1,53)	0.000	.92
	Calcium (mg)	−176.7	−121.9	0.899	5.81 (1,53)	0.101	.02
**Diet quality**							
	Healthy Eating Index- Canadian	26.5	6.9	0.655	27.34 (1,53)	0.345	<.001^d^
**Other outcomes**							
	Health-related quality of life short form-8	7.0	5.3	0.914	4.92 (1,53)	0.086	.03
	General Self-Efficacy	0.9	0.1	0.975	0.01 (1,53)	0.001	.27

^a^Exact statistic.

^b^g: gram.

^c^mg: milligram.

^d^Significant interactions of time by groups.

^e^DFE: dietary folate equivalent.

^f^mcg: microgram.

^g^IU: international unit.

### Qualitative Analysis

On the basis of analysis of textual data from the Web-based questionnaires, the personal goals that participants set for themselves tended to be related to weight loss; reducing sugar, fat, and processed food intakes; and increasing consumption of fruits and vegetables. In total, 5 focus groups (with a range of 5 to 9 participants) were conducted that generated more than 36,000 words of textual data. From the analysis, 3 categories of feedback emerged: (1) interpretation of nutrition-related gene test information, (2) facilitation of eating behavior change, and 3) directives for personalized nutrition practice.

#### Category 1: Interpretation of Nutrition-Related Gene Test Information

Participants from both groups thought richly detailed reports containing their nutrition-related gene test results were valuable:

...I think this is hugely valuable I really like this and I think you know inflammation seems to be a very big problem for a lot of people that could definitely help you find the right proteins the right fits whatever it is that it's going to assist your body in lowering that level and the next one hopefully having less disease or less arthritis or issues.

I think it was the detail. I was like “Wow” because I am scrolling down and I am like there is more and more. And you know, kind of did a cursory look and then I started to look more and I was like “Wow” because you know it really gets into specifics.

I loved it. It's well designed and well-structured so it's pretty good. Color coding was really great and you can easily get it just by a glance and see exactly what you are looking at.

Both groups shared insights about challenges with interpreting their results. Some C-group participants (n=4; received report only) tried to find a practitioner to review the results, which lead to variable responses:

I tried to hire a dietitian or nutritionist and I told them I had the DNA results and I said, “Are you willing to look at them and making a meal plan?” And they both said no.

Umm well my first impression was that some of the recommendations seemed to be non specific to but I just filtered through those and focused in on the ones that I thought were those were clearly for me...

...there are ideas in there but I needed it to be spelled out and I was willing to pay someone but I just couldn't find anyone to do it who was willing. That's the only thing.

...they {results} also gave me an opportunity to sit down with my Dr and talked about what I’ve been feeling and what these results and let's play with this and so she thought this was awesome. She thought this was really good she said...She’s very innovative and forward thinking.

#### Category 2: Facilitation of Eating Behavior Change

The participants revealed different components of their diet they either felt motivated to act on or that they took action on, regardless of which group they were assigned to. They expressed that the tailoring of information specific to their individual circumstances was motivating:

I think it made me feel more empowered. You just have more information.

In the past, I would be making all my selections and choices based on the general knowledge of a good lifestyle but now I know more about me and so I make personal choices. Knowing more about yourself and your body and what choices you make would be beneficial for you and what would be the most useful.

...I increased my protein and tried to make sure to incorporate some protein every meal and instead of just going for a quick grab of carb stuff I'd made sure that there was a balance, there was a protein...

...orange vegetables too so I’m more conscious about going after those as well and processing of the proteins I’m consuming too...

For those in the I group, the addition of the personalized nutrition report and dietitian consult that included review of nutrient analysis information from food records was reported to be helpful in selecting dietary intake targets to change:

It's amazing that the information that [dietitian’s name] gave me just learned about different oils and how they interact...

...I think that having [dietitian’s name] discuss it hit home more and it makes me more conscious, more aware. Yeah it also helps your understanding level because sometimes you look at things and your brain doesn’t connect with it the same way as having [dietitian’s name] say “well if you did this or that” and then your brain goes “oh okay, I can get that” and then you commit to it better.

I think it was fantastic especially if you are doing a collaboration with someone you could physically talk to and it would be more meaningful...having somebody to actually talk to and work through it together and say, “Okay, what does this all mean?” or “How does this all look?” really validates and reinforces the information that is contained in the book.

I think it did some motivation for sure, but I am not going to say 100% because it's not like I changed my whole [lifestyle]. You know, I choose salad over my French fries most of the week...I wouldn't say it was the genetic report that made me change this all, but it was the food record that made me realize it was out of whack.

...I think the benefits personally to me it was also being able to sit down and talk to [dietitian’s name] yes in relation to the report...

I didn’t realize how many hidden fats I was eating with the curries and all that but writing it down on paper and then have to have a talk about it. And the recommendation try to replace the fats with proteins so I am more likely to reach for the nuts than anything else...

#### Category 3: Directives for Future Personalized Nutrition Practice

Consistent with previous literature [13-15], many participants stated factors such as cost and privacy concerns were potential barriers to having nutrition-related gene testing done. Others within the I group noted that ongoing reinforcement was particularly helpful to them when trying to take action on the results. Most participants in both the I and C groups discussed that the major nutrients and salt were their key targets as the advice was easy for them to remember and follow; food components that were frequently mentioned in the focus groups by both the I and C groups included fat, caffeine, and lactose. Many indicated that trying to translate gene-based micronutrient results and the associated food-based recommendations into their daily diet was much more difficult:

But it didn't kick in again when I got the reminder on the survey and because I felt lost when we were left on our own whether it was sticking to it or not...And then we got the reminder, oh yeah yeah yes I did oranges...So they kicked me back into action...

I knew the vitamins one was probably the least influential on me and of least interest to me because I couldn’t wrap my head around how much all of these different types of vitamins that I am consuming and whether or not if I truly understand what each one what it’s purpose is and how it serves me. So I probably spent the least amount of time trying to, you know, contemplate and reflect on those results.

## Discussion

### Principal Findings

This study compared the effectiveness of standard and tailored gene-based personalized nutrition interventions in improving knowledge and motivation to change eating behavior, dietary intakes, and quality of life. A second objective was to elicit participant feedback from focus groups to better understand the quantitative findings. The results indicated that over a 9-week period, tailored interventions contributed to improved fat and sodium intakes, overall diet quality, and quality of life. In addition, the participants receiving these types of interventions typically indicated that they intended to make changes to their diet in the near future. When phenotypic plus genotypic information by group assignment was considered, improved total fat, saturated fat, and sodium intakes were found among those who possessed the risk genotype and received tailored dietary advice. Qualitative findings revealed that participants thought that the gene-based nutrition information motivated them to change eating behaviors. However, many indicated they found some of the information complicated, particularly the translation of micronutrient-based results and associated food-based recommended actions.

### Results Compared With Existing Research Literature

This study’s findings were consistent with other investigations that showed greater improvements in diet quality after receiving personalized practitioner-facilitated nutrition interventions when compared with receiving a standard gene test report [16-19,44]. Our findings indicated that at 6 weeks postintervention, many participants indicated that the DNA information related to diet continued to motivate them and to take action on healthy eating. However, the proportion of individuals reporting this declined over the course of the study indicating that ongoing practitioner-led reinforcement may be more helpful in keeping participants focused on continued action to eat healthier. Similar to other studies, when genotypic and phenotypic information was considered by group assignment, significant positive differences in select nutrient intakes occurred [44]. These findings that demonstrate that the provision of nutrition-based genetic information improves dietary behaviors are practices that can facilitate eating behavior changes [45]; however, further research is needed to determine their generalizability to other populations and if the improvements are sustained long term.

The results that suggested receiving gene test results motivated participants to change their diet have also been reported in other studies [46]. In addition, the qualitative findings suggested that for many participants their main diet-related goal after receiving the results was weight management. Research suggests that motivation to positively change eating behaviors is also based on factors such as ethical concerns and mood. If a practitioner has previous knowledge of what people’s motives are, they can use this information combined with gene test results to develop appropriate goals and further inspire them to make positive dietary changes [19].

Based on the experience of other investigators [16-19], the direction in dietary changes in relation to the nutrition-based *risk* variant education provided was not always consistent, and in some instances, they were counterintuitive. An example of this included the significant declines in MDs of intake for calcium and vitamin B_12_. It is thought these decreases may have been because of reductions in intake of sources of saturated fat (eg, meat and milk-based products). Alternatively, as found in the Food4Me Study [18], if people were informed that they had the nonrisk version of the genotype, they may not be motivated to focus on the associated dietary component. The findings that showed declines in folate intake MDs are consistent with other findings, which suggest that regardless of whether one is told they do or do not have the risk version of the *MTHFR 677CT* gene, the information does not seem to translate into meaningful differences in folate intake measures [47]. On the basis of insights from our qualitative data, the variable findings among intakes of vitamin and mineral intakes may be because of a lack of understanding by the participant of their functions and relevance. Clearly, future work is needed to better understand how the presentation of information for different genotypes can be better used to improve a targeted eating behavior.

To the best of our knowledge, the findings that showed time-by-group interactions for sodium and overall diet quality have not been reported elsewhere. In particular, the I group had more pronounced reductions in dietary sodium intake compared with the C group. These results have implications for the prevention and treatment of hypertension, a condition that can be exacerbated by high sodium intakes and can progress to coronary heart disease in some individuals. Hypertension is becoming increasingly prevalent, affecting an estimated 1.13 billion people worldwide [48,49]. Hypertension and coronary heart disease are related to both genetic predisposition and environmental exposures such as an unhealthy diet. This study’s findings imply that tailored nutritional advisement could help prevent epigenetic changes that contribute to the development and progression of these chronic conditions. As a result, substantial savings in health care costs could be realized.

The findings that indicated that significantly greater improvements in quality-of-life measures occurred in the I group were novel. Although previous studies have shown relationships between poorer quality diet and worse quality of life [50], to the best of our knowledge, this is the first time this has been reported related to gene-based personalized nutrition interventions. We thought that GSE might play a role in this relationship; however, when we separately analyzed dietary quality as a dependent variable and quality-of-life scores as the independent variable and controlled for GSE, the findings were nonsignificant.

It was surprising that dietary improvements were not observed across all results where nutrition-related risk variants were present in the participants. The focus group data helped to provide insights about these findings by suggesting that although participants found the information useful, it was also overwhelming. As a result, they would try to simplify the implementation of their results by adjusting intakes of the major nutrients and sodium and pay less attention to the micronutrients-based gene information. However, in doing this, they may not be substantially improving the overall quality of their diet or meeting all of their micronutrient requirements. For the future, improved nutrition education strategies are needed that will facilitate the uptake of all recommended dietary changes that are based on gene test results.

### Strengths and Limitations

This study’s strengths include its high retention rate, focus on a defined adult population, assessment of dietary intakes using FFQ estimates and 3-day food records to reduce misreporting error, and the provision of quantitative and qualitative data. However, this investigation could have been strengthened by including objective measures such as biochemical indicators of nutrient status. The modest sample size prevented stratification of results based on individual genes. Furthermore, given the composition of the sample was mainly female and Caucasian, the generalizability of the results is limited.

### Implications and Conclusions

Providing participants with DNA information related to diet improved knowledge, motivation, and action related to healthy eating. However, tailored practitioner-led, gene-based personalized nutrition interventions tend to be more effective in improving dietary intakes of key target nutrients such as fat and sodium. Further work is needed to produce more comprehensive changes in dietary intakes that are based on gene test results. The study results will be leveraged to generate new, tailored, and digitally based nutrigenomics education tools that may help to advance gene-based personalized nutrition practice.

## Multimedia Appendix 1

Participant exclusion criteria.

## Multimedia Appendix 2

Knowledge, attitudes, and behavior baseline to study completion. Stages of change: proportion who changed throughout the study.

## Multimedia Appendix 3

CONSORT‐EHEALTH checklist (V 1.6.2).

## Abbreviations

C: control group
DFE: dietary folate equivalent
FFQ: Food Frequency Questionnaire
GSE: General Self-Efficacy
I: intervention group
HEI-C: Healthy Eating Index–Canadian
HRQoL: health-related quality of life
MD: mean difference
NHP: natural health product
RD: registered dietitian

## References

[1] Simopoulos AP (2010). Nutrigenetics/Nutrigenomics. Annu Rev Public Health.

[2] De Caterina R (2011). Genetic and nutritional interactions in cardiovascular disease. World Rev Nutr Diet.

[3] Tammen SA, Friso S, Choi S (2013). Epigenetics: the link between nature and nurture. Mol Aspects Med.

[4] Berná G, Oliveras-López MJ, Jurado-Ruíz E, Tejedo J, Bedoya F, Soria B, Martín F (2014). Nutrigenetics and nutrigenomics insights into diabetes etiopathogenesis. Nutrients.

[5] Mathers JC (2017). Nutrigenomics in the modern era. Proc Nutr Soc.

[6] El-Sohemy A, Cornelis MC, Kabagambe EK, Campos H (2007). Coffee, CYP1A2 genotype and risk of myocardial infarction. Genes Nutr.

[7] Palatini P, Ceolotto G, Ragazzo F, Dorigatti F, Saladini F, Papparella I, Mos L, Zanata G, Santonastaso M (2009). CYP1A2 genotype modifies the association between coffee intake and the risk of hypertension. J Hypertens.

[8] Solis C, Veenema K, Ivanov AA, Tran S, Li R, Wang W, Moriarty DJ, Maletz CV, Caudill MA (2008). Folate intake at RDA levels is inadequate for Mexican American men with the methylenetetrahydrofolate reductase 677TT genotype. J Nutr.

[9] Guinotte CL, Burns MG, Axume JA, Hata H, Urrutia TF, Alamilla A, McCabe D, Singgih A, Cogger EA, Caudill MA (2003). Methylenetetrahydrofolate reductase 677C-->T variant modulates folate status response to controlled folate intakes in young women. J Nutr.

[10] Tsang BL, Devine OJ, Cordero AM, Marchetta CM, Mulinare J, Mersereau P, Guo J, Qi YP, Berry RJ, Rosenthal J, Crider KS, Hamner HC (2015). Assessing the association between the methylenetetrahydrofolate reductase (MTHFR) 677C>T polymorphism and blood folate concentrations: a systematic review and meta-analysis of trials and observational studies. Am J Clin Nutr.

[11] Ferguson JF, Phillips CM, McMonagle J, Pérez-Martínez P, Shaw DI, Lovegrove JA, Helal O, Defoort C, Gjelstad IMF, Drevon CA, Blaak EE, Saris WHM, Leszczyńska-Gołabek I, Kiec-Wilk B, Risérus U, Karlström B, Lopez-Miranda J, Roche HM (2010). NOS3 gene polymorphisms are associated with risk markers of cardiovascular disease, and interact with omega-3 polyunsaturated fatty acids. Atherosclerosis.

[12] Poch E, González D, Giner V, Bragulat E, Coca A, de La Sierra A (2001). Molecular basis of salt sensitivity in human hypertension. Evaluation of renin-angiotensin-aldosterone system gene polymorphisms. Hypertension.

[13] Edwards KT, Huang CJ (2014). Bridging the consumer-medical divide: how to regulate direct-to-consumer genetic testing. Hastings Cent Rep.

[14] Covolo L, Rubinelli S, Ceretti E, Gelatti U (2015). Internet-based direct-to-consumer genetic testing: a systematic review. J Med Internet Res.

[15] Goldsmith L, Jackson L, O'Connor A, Skirton H (2013). Direct-to-consumer genomic testing from the perspective of the health professional: a systematic review of the literature. J Community Genet.

[16] Nielsen DE, El-Sohemy A (2014). Disclosure of genetic information and change in dietary intake: a randomized controlled trial. PLoS One.

[17] Livingstone KM, Celis-Morales C, Navas-Carretero S, San-Cristobal R, Macready AL, Fallaize R, Forster H, Woolhead C, O'Donovan CB, Marsaux CF, Kolossa S, Tsirigoti L, Lambrinou CP, Moschonis G, Godlewska M, Surwiłło A, Drevon CA, Manios Y, Traczyk I, Gibney ER, Brennan L, Walsh MC, Lovegrove JA, Saris WH, Daniel H, Gibney M, Martinez JA, Mathers JC, Food4Me Study (2016). Effect of an internet-based, personalized nutrition randomized trial on dietary changes associated with the Mediterranean diet: the Food4Me Study. Am J Clin Nutr.

[18] Fallaize R, Celis-Morales C, Macready AL, Marsaux CF, Forster H, O'Donovan C, Woolhead C, San-Cristobal R, Kolossa S, Hallmann J, Mavrogianni C, Surwillo A, Livingstone KM, Moschonis G, Navas-Carretero S, Walsh MC, Gibney ER, Brennan L, Bouwman J, Grimaldi K, Manios Y, Traczyk I, Drevon CA, Martinez JA, Daniel H, Saris WH, Gibney MJ, Mathers JC, Lovegrove JA, Food4Me Study (2016). The effect of the apolipoprotein E genotype on response to personalized dietary advice intervention: findings from the Food4Me randomized controlled trial. Am J Clin Nutr.

[19] O'Donovan CB, Walsh MC, Gibney MJ, Brennan L, Gibney ER (2017). Knowing your genes: does this impact behaviour change?. Proc Nutr Soc.

[20] Celis-Morales C, Marsaux CF, Livingstone KM, Navas-Carretero S, San-Cristobal R, Fallaize R, Macready AL, O'Donovan C, Woolhead C, Forster H, Kolossa S, Daniel H, Moschonis G, Mavrogianni C, Manios Y, Surwillo A, Traczyk I, Drevon CA, Grimaldi K, Bouwman J, Gibney MJ, Walsh MC, Gibney ER, Brennan L, Lovegrove JA, Martinez JA, Saris WH, Mathers JC (2017). Can genetic-based advice help you lose weight? Findings from the Food4Me European randomized controlled trial. Am J Clin Nutr.

[21] Littlejohn P, Cop I, Brown E, Afroze R, Davison KM (2018). Comparison of nutrigenomics technology interface tools for consumers and health professionals: protocol for a mixed-methods study. JMIR Res Protoc.

[22] Institute of Medicine (2001). Dietary Reference Intakes: Applications in Dietary Assessment.

[23] Health Canada (2007). Eating Well With Canada's Food Guide.

[24] British Columbia Government (2004). British Columbia Nutrition Survey - Report on Food Group Use.

[25] Grimaldi KA, van Ommen B, Ordovas JM, Parnell LD, Mathers JC, Bendik I, Brennan L, Celis-Morales C, Cirillo E, Daniel H, de Kok B, El-Sohemy A, Fairweather-Tait SJ, Fallaize R, Fenech M, Ferguson LR, Gibney ER, Gibney M, Gjelstad IMF, Kaput J, Karlsen AS, Kolossa S, Lovegrove J, Macready AL, Marsaux CFM, Alfredo MJ, Milagro F, Navas-Carretero S, Roche HM, Saris WHM, Traczyk I, van Kranen H, Verschuren L, Virgili F, Weber P, Bouwman J (2017). Proposed guidelines to evaluate scientific validity and evidence for genotype-based dietary advice. Genes Nutr.

[26] (2017). SurveyMonkey Inc.

[27] Eysenbach G (2004). Improving the quality of Web surveys: the Checklist for Reporting Results of Internet E-Surveys (CHERRIES). J Med Internet Res.

[28] Health Canada (1999). BC Nutrition Survey Training Manual.

[29] Garriguet D (2009). Diet quality in Canada. Health Rep.

[30] Lowe MR (1993). The effects of dieting on eating behavior: a three-factor model. Psychol Bull.

[31] Turner-Bowker DM, Bayliss MS, Ware JE, Kosinski M (2003). Usefulness of the SF-8 Health Survey for comparing the impact of migraine and other conditions. Qual Life Res.

[32] Ware J, Kosinski M, Dewey J, Gandek B (2001). How to Score and Interpret Single-Item Health Status Measures: A Manual for Users of the SF-8 Health Survey.

[33] Schwarzer R, Jerusalem M, Weinman J, Wright S, Johnston M (1995). Generalized Self-Efficacy Scale. Measures in health psychology: A User's Portfolio. Causal and Control Beliefs.

[34] Luszczynska A, Scholz U, Schwarzer R (2005). The general self-efficacy scale: multicultural validation studies. J Psychol.

[35] Prochaska JO, DiClemente CC (1992). Stages of change in the modification of problem behaviors. Prog Behav Modif.

[36] Taylor HL, Jacobs DR, Schucker B, Knudsen J, Leon AS, Debacker G (1978). A questionnaire for the assessment of leisure time physical activities. J Chronic Dis.

[37] Statistics Canada (2010). Canadian Community Health Survey.

[38] Yu L, Buysse DJ, Germain A, Moul DE, Stover A, Dodds NE, Johnston KL, Pilkonis PA (2011). Development of short forms from the PROMIS™ sleep disturbance and sleep-related impairment item banks. Behav Sleep Med.

[39] (2017). ESHA Research: Food Labeling Software | Nutrition Analysis Software.

[40] Health Canada (2016). Canadian Nutrient File.

[41] Stata Corporation (2011). Stata Statistical Software: Release 2.

[42] (2015). NVivo qualitative data analysis software, Version 11.

[43] Sandelowski M (1995). Qualitative analysis: what it is and how to begin. Res Nurs Health.

[44] Forster H, Walsh MC, O'Donovan CB, Woolhead C, McGirr C, Daly EJ, O'Riordan R, Celis-Morales C, Fallaize R, Macready AL, Marsaux CF, Navas-Carretero S, San-Cristobal R, Kolossa S, Hartwig K, Mavrogianni C, Tsirigoti L, Lambrinou CP, Godlewska M, Surwiłło A, Gjelstad IM, Drevon CA, Manios Y, Traczyk I, Martinez JA, Saris WH, Daniel H, Lovegrove JA, Mathers JC, Gibney MJ, Gibney ER, Brennan L (2016). A dietary feedback system for the delivery of consistent personalized dietary advice in the web-based multicenter Food4Me study. J Med Internet Res.

[45] Marteau TM, French DP, Griffin SJ, Prevost AT, Sutton S, Watkinson C, Attwood S, Hollands GJ (2010). Effects of communicating DNA-based disease risk estimates on risk-reducing behaviours. Cochrane Database Syst Rev.

[46] Rankin A, Bunting BP, Poínhos R, van der Lans IA, Fischer AR, Kuznesof S, Almeida M, Markovina J, Frewer LJ, Stewart-Knox BJ (2018). Food choice motives, attitude towards and intention to adopt personalised nutrition. Public Health Nutr.

[47] O'Donovan CB, Walsh MC, Forster H, Woolhead C, Celis-Morales C, Fallaize R, Macready AL, Marsaux CF, Navas-Carretero S, San-Cristobal R, Kolossa S, Mavrogianni C, Lambrinou CP, Moschonis G, Godlewska M, Surwillo A, Bouwman J, Grimaldi K, Traczyk I, Drevon CA, Daniel H, Manios Y, Martinez JA, Saris WH, Lovegrove JA, Mathers JC, Gibney MJ, Brennan L, Gibney ER (2016). The impact of 677C → T risk knowledge on changes in folate intake: findings from the Food4Me study. Genes Nutr.

[48] NCD Risk Factor Collaboration (NCD-RisC) (2017). Worldwide trends in blood pressure from 1975 to 2015: a pooled analysis of 1479 population-based measurement studies with 19·1 million participants. Lancet.

[49] (2015). Global Health Observatory (GHO) data.

[50] Jing X, Chen J, Dong Y, Han D, Zhao H, Wang X, Gao F, Li C, Cui Z, Liu Y, Ma J (2018). Related factors of quality of life of type 2 diabetes patients: a systematic review and meta-analysis. Health Qual Life Outcomes.

