# Supplementary material for: Comparison of Nutrigenomics Technology Interface Tools for Consumers and Health Professionals: A Sequential Explanatory Mixed Methods Investigation
Source: J Med Internet Res. 2019 Jun 28;21(6):e12580. doi: 10.2196/12580 (PMC6625748; doi:10.2196/12580)
Supplement: Supplementary file 1 [file jmir_v21i6e12580_app1.doc]

| **Multimedia Appendix 1: Participant exclusion criteria** |
| --- |
| - 1. Currently following a therapeutic or restrictive diet.   2. Diagnosis of ≥ 2 chronic diseases or an unstable chronic disease as deemed by accepted clinical guidelines.   3. Any of the following conditions: HIV; chronic obstructive pulmonary disease; severe/uncontrolled asthma; cystic fibrosis; bronchiectasis; interstitial lung disease; chronic renal failure; colon or small intestine problem; liver or kidney disease; uncorrected hypothyroidism or hyperthyroidism, alcohol or drug dependence during previous 12 months; current or former malignancy for which the participant has undergone resection, radiation therapy or chemotherapy within previous 5 years.   4. Currently enrolled or plan to be enrolled in another research study during the course of the investigation.   5. Planned or recent (within the last 12 months) bariatric surgery.   6. Current use of weight altering medication for the purpose of weight loss.   7. Investigators and their immediate families (ie, spouse, parent, child or sibling, whether biological or legally adopted).   8. Pregnant and/or breastfeeding.   9. Current smoker.   10. BMI ≥ 35.   11. Any other health risk or condition that may put the participant at risk, influence the results of the study, or the ability to participate in the study. |
