# Supplementary material for: Comparison of Nutrigenomics Technology Interface Tools for Consumers and Health Professionals: A Sequential Explanatory Mixed Methods Investigation
Source: J Med Internet Res. 2019 Jun 28;21(6):e12580. doi: 10.2196/12580 (PMC6625748; doi:10.2196/12580)
Supplement: Supplementary file 2 [file jmir_v21i6e12580_app2.doc]

| Supplementary File 2: Knowledge, Attitudes and Behavior Baseline to Study Completion | | | | | | |
| --- | --- | --- | --- | --- | --- | --- |
| 1. Baseline Assessment | | | | | | |
| Question (baseline questionnaire) | | | % Agree (n) | | | |
| I believe my DNA information related to diet will: | | | | | | |
| Help me to UNDERSTAND my health better. | | | 98% (54) | | | |
| MOTIVATE me to become more conscious about healthy eating for my body. | | | 95% (52) | | | |
| Help me to TAKE ACTION to eat healthier for my body. | | | 93% (51) | | | |
| **2. 3 Weeks Post Intervention Questions to Control and Intervention Group** | | | | | | |
| Question | Group | % Agree (n) | | *P* -value | | |
| Knowing my DNA information related to diet is: | | | | | | |
| Helping me to UNDERSTAND my health better. | I | 89% (32) | | .702 | | |
|  | C | 84% (16) | |  |  |  |
| MOTIVATING me to be more conscious about healthy eating for my body. | I | 87% (31) | | .322 | | |
|  | C | 75% (14) | |  |  |  |
| Helping me to TAKE ACTION to eat healthier for my body. | I | 87% (31) | | .161 | | |
|  | C | 68% (13) | |  |  |  |
| 3. 6 Weeks Post Intervention Questions to Control and Intervention Group | | | | | | |
| Question | Group | % Agree (n) | | | *P* -value | |
| Knowing my DNA information related to diet is: |  |  | | |  | |
| Helping me to UNDERSTAND my health better. | I | 54% (19) | | | .915 | |
|  | C | 32% (6) | | |  |  |
| MOTIVATING me to be more conscious about healthy eating for my body. | I | 54% (19) | | | .575 | |
|  | C | 35% (7) | | |  |  |
| Helping me to TAKE ACTION to eat healthier for my body. | I | 57% (20) | | | .272 | |
|  | C | 30% (6) | | |  |  |
| Helping me with specific foods and meals to eat healthier for my body. | I | 63% (23) | | | -- | |
|  | C | 4% (10) | | |  |  |
| Since getting my DNA information related to diet, I am taking action on: | | | | | | |
| Reading nutrition labels more often | I | 49% (17) | | | | .470 |
|  | C | 32% (6) | | | |  |
| Selecting healthier food choices at restaurants | I | 57% (20) | | | | .867 |
|  | C | 35% (7) | | | |  |
| Purchasing healthier food (e.g., buying fresh fruits & vegetables) | I | 51% (19) | | | | .423 |
|  | C | 27% (5) | | | |  |
| Making healthier meals at home (e.g., less packaged, processed foods) | I | 50% (18) | | | | .982 |
|  | C | 31% (6) | | | |  |
| Taking supplements to support my DNA | I | 38% (14) | | | | .286 |
|  | C | 16% (3) | | | |  |
| Adjusting my eating to my DNA | I | 49% (17) | | | | .982 |
|  | C | 30% (6) | | | |  |

^a^Statistic cannot be computed due to at least one cell having a frequency count of 0

I=Intervention (n=36); C= Control (n=19)

| **Supplementary File 3: Stages of Change: Proportion Who Changed Throughout Study** | | | | | | |
| --- | --- | --- | --- | --- | --- | --- |
| Variable | Group | Baseline | 3 weeks | | 6 weeks | |
|  |  | % Agree (n) | % Agree (n) | *P* -value^a^ | % Agree (n) | *P* -value^a^ |
| Outside of this research study, please tell me which of the following best describes you? | | | | | | |
| I personally do not have any immediate plans to eat a healthy diet. | I | 53% (19) | 80% (29) | .0001 | 0% (0) | --^b^ |
|  | C | 89% (17) | 79% (15) | .4252 | 0% (0) | --^b^ |
| I am thinking about making healthy changes to my diet within the next 6 months. | I | 82% (30) | 100% (36) | .2272 | 61% (22) | .090 |
|  | C | 94% (18) | 100% (19) | .1724 | 79% (15) | .199 |
| I plan to make healthy changes to my diet within the next month. | I | 86% (31) | 48% (17) | .0023 | 57% (20) | .017 |
|  | C | 93% (18) | 21% (4) | .0000 | 86% (16) | .501 |
| I generally eat a healthy diet (eg, at least 3-5 servings of fruits and vegetables). | I | 64% (23) | 65% (23) | .9273 | 78% (28) | .242 |
|  | C | 63% (12) | 89% (17) | .0702 | 79% (15) | .338 |
| I have been eating a healthy diet for more than 12 months. | I | 48% (17) | 70% (25) | .0828 | 57% (20) | .554 |
|  | C | 42% (8) | 79% (15) | .0202 | 36% (7) | .710 |

^a^Test of proportions based on agreement with statement and compared to baseline measures

^b^Statistic cannot be computed due to table cells having frequencies of 0

I=Intervention (n=36); C= Control (n=19)
