# Supplementary material for: Comparison of Nutrigenomics Technology Interface Tools for Consumers and Health Professionals: A Sequential Explanatory Mixed Methods Investigation
Source: J Med Internet Res. 2019 Jun 28;21(6):e12580. doi: 10.2196/12580 (PMC6625748; doi:10.2196/12580)
Supplement: Supplementary file 3 [file jmir_v21i6e12580_app3.pdf]

CONSORT-EHEALTH Checklist V1.6.2 Report	Manuscript Number	12580
**(based on CONSORT-EHEALTH V1.6), available at [http://tinyurl.com/consort-ehealth-v1-6].**		
**Date completed**		
6/22/2019 2:37:23		
**by**		
Karen Davison		
Comparison of Nutrigenomics Technology Interface Tools for Consumers and Health Professionals: A Sequential Explanatory Mixed Methods Investigation		
TITLE		
**1a-i) Identify the mode of delivery in the title**		
Comparison of nutrigenomics technology interface tools for consumers and health professionals: a sequential explanatory mixed methods investigation		
**1a-ii) Non-web-based components or important co-interventions in title**		
No		
**1a-iii) Primary condition or target group in the title**		
health professionals, consumers		
ABSTRACT		
**1b-i) Key features/functionalities/components of the intervention and comparator in the METHODS section of the ABSTRACT**		
Sequential explanatory mixed methods investigation of 55 healthy adults (35-55 years) was conducted that included: 1) a 9-week RCT where participants were randomized to receive a standard nutrition-based gene test report (control; n=19) or a practitioner-facilitated personalized nutrition intervention integrating gene test results, health assessment data, and participant goals (intervention; n=36)		
**1b-ii) Level of human involvement in the METHODS section of the ABSTRACT**		
Sequential explanatory mixed methods investigation of 55 healthy adults (35-55 years) was conducted that included: 1) a 9-week RCT where participants were randomized to receive a standard nutrition-based gene test report (control; n=19) or a practitioner-facilitated personalized nutrition intervention integrating gene test results, health assessment data, and participant goals (intervention; n=36)		
**1b-iii) Open vs. closed, web-based (self-assessment) vs. face-to-face assessments in the METHODS section of the ABSTRACT**		
Sequential explanatory mixed methods investigation of 55 healthy adults (35-55 years) was conducted that included: 1) a 9-week RCT where participants were randomized to receive a standard nutrition-based gene test report (control; n=19) or a practitioner-facilitated personalized nutrition intervention integrating gene test results, health assessment data, and participant goals (intervention; n=36)		
**1b-iv) RESULTS section in abstract must contain use data**		
Of the 55 (55/58 enrolled, 95%) participants who completed the study, most were between 40 to 51 years (n=37; 67%), female (n=41; 75%), in a relationship (n=47; 86%), completed post-secondary education (n=34; 62%), and earned a high household income (n=32; 58%).		
**1b-v) CONCLUSIONS/DISCUSSION in abstract for negative trials**		
While improvements were observed in both groups, healthy adults appear to derive better dietary outcomes and perceived health bene□ts from practitioner-led personalized nutrition interventions. In some instances, combining genotypic and phenotypic information facilitates positive dietary changes.		
INTRODUCTION		
**2a-i) Problem and the type of system/solution**		

Although the advancement of nutrigenomics-based personalized nutrition shows signifcant promise in improving population health, it also presents challenges. These issues include concerns about the complexity in translating gene-based results into meaningful recommendations that will lead to positive health outcomes [13-15]. Differences in dietary intakes are not always observed between ``risk`` and ``nonrisk`` groups [20] and dietary changes have not been consistently observed across all identified risk gene variants (e.g., MTHFR) where nutrition advice is provided [16-19]. In order for nutrigenomics and personalized nutrition to advance in health practice, better interface educational tools (e.g., web applications, targeted messaging after personalized nutrition advice provided) need to be developed that are easily implemented by practitioners, understood by consumers, and that foster positive eating behavior changes. Furthermore, they need to incorporate accepted nutrition guidelines, integrate phenotypic information about current health status, and align with behavior change theory principles [5, 20].		
**2a-ii) Scientific background, rationale: What is known about the (type of) system**		
Since the success of the Human Genome project, science technology has advanced rapidly in several disciplines, including medicine and nutrition. Given that the interaction of nutrients with DNA can impact nutritional status and the development of complex diseases, nutritional genomics (nutrigenomics) has become an increasingly important in nutrition practice. Nutrigenomics encompasses nutrigenetics, which investigates the effect of genetic variation on nutrient bioavailability and metabolism, and nutrigenomics, which examines how nutrients and bioactive food compounds affect human health through epigenetic modifications [1-5].		
**Does your paper address CONSORT subitem 2b?**		
The main study objectives were to compare a practitioner-facilitated personalized dietary approach that uses genotypic and phenotypic information to a self-driven approach and their impacts on changing participant’s knowledge, motivation, and behavior related to eating habits and the quality of their diet. It was hypothesized that signifcantly higher levels of knowledge, motivation, and behavior would be reported and that there would be a higher level of diet quality changes in the group that receives personal DNA diet information plus customized dietary advice (practitioner led) compared to the group that is provided personal DNA diet information (direct-to-consumer self- driven approach) only. In addition, self-effcacy and quality of life measures were evaluated as potential mediators/moderators of dietary changes and outcomes.		
METHODS		
**3a) CONSORT: Description of trial design (such as parallel, factorial) including allocation ratio**		
The sequential explanatory mixed methods investigation consisted of: 1) a randomized controlled trial (RCT: 2:1 allocation ratio) comparing standard selfdriven versus practitioner-facilitated approaches that use DNA-based diet information; and 2) qualitative investigation of participants’ experiences to help interpret the intervention’s quantitative outcomes.		
**3b) CONSORT: Important changes to methods after trial commencement (such as eligibility criteria), with reasons**		
The study protocol, including paper-based on online data collection forms, was approved by Quorum Institutional Review Board (protocol # 32220CDN/1). No changes were made		
**3b-i) Bug fixes, Downtimes, Content Changes**		
Not applicable		
**4a) CONSORT: Eligibility criteria for participants**		
Yes - information provided in multimedia appendex		
**4a-i) Computer / Internet literacy**		
Eligibility criteria included that they wanted to improve their health, could understand and provide informed consent, and were willing to provide a buccal swab for DNA testing. Exclusion criteria are specified in (Multimedia appendix 1. Supplementary file 1.		
**4a-ii) Open vs. closed, web-based vs. face-to-face assessments:**		
Participants selected for the study included healthy medically stable adults (35-55 years) residing in the greater Vancouver area of the province of British Columbia, Canada. Participants were recruited via social media, a newspaper article, and posters.		

**4a-iii) Information giving during recruitment**		
All participants provided initial online consent to collect eligibility screening<br>information and, if eligible, baseline information related to their health. At the<br>participant’s first site visit, a second written consent form was reviewed which<br>participants signed to confirm their continued involvement in the study.		
**4b) CONSORT: Settings and locations where the data were collected**		
Participants selected for the study included healthy medically stable adults (35-55 years) residing in the greater Vancouver area of the province of British Columbia, Canada. Participants were recruited via social media, a newspaper article, and posters.		
**4b-i) Report if outcomes were (self-)assessed through online questionnaires**		
Recruiting/Screening: This included the initial eligibility screen and, where<br>applicable, baseline assessment (online) that collected information about<br>sociodemographics, current health status (e.g., presence of health conditions, medication and supplement usage), quality of life, self-e□cacy, questions about knowledge, motivation, and action related to DNA-based information, stage of change, physical and sedentary activities, food intakes (food frequency, food selection), anthropometrics, and sleep quality.<br>4. Follow-up #1. An online survey was sent to I and C participants at week 3<br>postintervention to collect data about any changes in income, social support, changes in<br>knowledge, behavior, and action, stage of change, and adverse event information.<br>For the I group, questions that asked about whether knowing one’s personal DNA<br>helped with eating behavior change were also included.<br>5. Follow-up #2. Six weeks after the intervention participants received the online<br>baseline health assessment questionnaire and food records to complete in<br>preparation for the final on-site visit (week 8 post-intervention).		
**4b-ii) Report how institutional affiliations are displayed**		
Not applicable		
**5) CONSORT: Describe the interventions for each group with sufficient details to allow replication, including how and when they were actually administered**		
**5-i) Mention names, credential, affiliations of the developers, sponsors, and owners**		
Not applicable		
**5-ii) Describe the history/development process**		
Not applicable		
**5-iii) Revisions and updating**		
Not applicable		
**5-iv) Quality assurance methods**		
Quantitative Analysis<br>Food Intake and Nutrient Analysis: Nutrient analysis was conducted using ESHA - The Food Processor Nutrition Analysis and Fitness software and the Canadian<br>Nutrient File [41, 42]. Averages of the three days of nutrient values were used in the analysis. FFQ values were used to derive usual intakes of the nutrients of interest [24]. All analyses were done on an intent-to-treat basis using<br>STATA software [43].<br>Qualitative Analysis<br>Textual data from the online questionnaires (e.g., participant’s personal dietary<br>goals) were grouped into categories where feasible. Data from the focus groups<br>were transcribed, entered into NVivo [44], and analyzed by research team members using interpretative thematic analysis to identify patterns, concepts, themes, and examples in relation to existing behavior change theories and the study objectives [45]. Interpretations were reviewed by research team members and participants to check for descriptive and interpretative validity. Qualitative data was reported based on thematic analysis derived from three independent reviews of the textual data.		

**5-v) Ensure replicability by publishing the source code, and/or providing screenshots/screen-capture video, and/or providing flowcharts of the algorithms used**		
Not applicable		
**5-vi) Digital preservation**		
Not applicable		
**5-vii) Access**		
Not applicable		
**5-viii) Mode of delivery, features/functionalities/components of the intervention and comparator, and the theoretical framework**		
Not applicable		
**5-ix) Describe use parameters**		
Not applicable		
**5-x) Clarify the level of human involvement**		
The sequential explanatory mixed methods investigation consisted of: 1) a<br>randomized controlled trial (RCT: 2:1 allocation ratio) comparing standard selfdriven versus practitioner-facilitated approaches that use DNA-based diet information; and 2) qualitative investigation of participants’ experiences to help<br>interpret the intervention’s quantitative outcomes.		
**5-xi) Report any prompts/reminders used**		
Not applicable		
**5-xii) Describe any co-interventions (incl. training/support)**		
Not applicable		
**6a) CONSORT: Completely defined pre-specified primary and secondary outcome measures, including how and when they were assessed**		
Nutrition-related Outcomes: All dietary intake data collection was done according to standard procedures [24, 28]. Three-day food records measured pre/post caloric,<br>macronutrient, micronutrient, and food group intakes which were compared to<br>nutrition standards. The Canadian version of the Healthy Eating Index (HEI-C) [29] was used to assess diet quality.<br>Health Related Quality of Life (HRQOL) SF-8 (Short Form 8): A validated<br>measurement tool of quality of life, functional health and well-being [32] based on a 4-week recall period.<br>General Self E□cacy (GSE): A validated item measure of self-e□cacy shown to<br>correlate with emotion, optimism and work satisfaction [34].<br>Measures of Change in Knowledge, Motivation, and Behavior: Three questions to<br>assess for changes in knowledge, motivation, and behavior related to DNA-based dietary advice were developed by the authors based on the Stages of Change Model [36] and current review of the evidence.		
**6a-i) Online questionnaires: describe if they were validated for online use and apply CHERRIES items to describe how the questionnaires were designed/deployed**		
FluidSurveys software [26] was used to construct the online closed questionnaires. The online questionnaires were developed using the Checklist for Reporting Results of Internet E-Surveys [27], standard measurements (detailed in measurements section), and protocols for nutrition assessment [25]. All questionnaires contained 12 or less screens (pages) and were pilot tested with study staff and student volunteers (n=11) to assess usability and technical functionality. Participants were emailed instructions and the links to each online questionnaire at the appropriate times during the study.		
**6a-ii) Describe whether and how “use” (including intensity of use/dosage) was defined/measured/monitored**		
Not applicable		
**6a-iii) Describe whether, how, and when qualitative feedback from participants was obtained**		

Textual data from the online questionnaires (e.g., participant's personal dietary<br>goals) were grouped into categories where feasible. Data from the focus groups<br>were transcribed, entered into NVivo [44], and analyzed by research team members using interpretative thematic analysis to identify patterns, concepts, themes, and examples in relation to existing behavior change theories and the study objectives [45]. Interpretations were reviewed by research team members and participants to check for descriptive and interpretative validity. Qualitative data was reported based on thematic analysis derived from three independent reviews of the textual data.		
**6b) CONSORT: Any changes to trial outcomes after the trial commenced, with reasons**		
Participants selected for the study included healthy medically stable adults (35-55 years) residing in the greater Vancouver area of the province of British Columbia, Canada. Participants were recruited via social media, a newspaper article, and posters.		
**7a) CONSORT: How sample size was determined**		
**7a-i) Describe whether and how expected attrition was taken into account when calculating the sample size**		
Detailed in protocol paper		
**7b) CONSORT: When applicable, explanation of any interim analyses and stopping guidelines**		
Nutrition-related Outcomes: All dietary intake data collection was done according to standard procedures [24, 28]. Three-day food records measured pre/post caloric,<br>macronutrient, micronutrient, and food group intakes which were compared to<br>nutrition standards. The Canadian version of the Healthy Eating Index (HEI-C) [29] was used to assess diet quality.<br>Health Related Quality of Life (HRQOL) SF-8 (Short Form 8): A validated<br>measurement tool of quality of life, functional health and well-being [32] based on a 4-week recall period.<br>General Self E□cacy (GSE): A validated item measure of self-e□cacy shown to<br>correlate with emotion, optimism and work satisfaction [34].<br>Measures of Change in Knowledge, Motivation, and Behavior: Three questions to<br>assess for changes in knowledge, motivation, and behavior related to DNA-based dietary advice were developed by the authors based on the Stages of Change Model [36] and current review of the evidence.		
**8a) CONSORT: Method used to generate the random allocation sequence**		
Not applicable		
**8b) CONSORT: Type of randomisation; details of any restriction (such as blocking and block size)**		
Participants were randomized, using a random number generator by a statistician independent to the study into either the intervention or control group.		
**9) CONSORT: Mechanism used to implement the random allocation sequence (such as sequentially numbered containers), describing any steps taken to conceal the sequence until interventions were assigned**		
Participants were randomized, using a random number generator by a statistician independent to the study into either the intervention or control group.		
**10) CONSORT: Who generated the random allocation sequence, who enrolled participants, and who assigned participants to interventions**		
Statistician		
**11a) CONSORT: Blinding - If done, who was blinded after assignment to interventions (for example, participants, care providers, those assessing outcomes) and how**		
**11a-i) Specify who was blinded, and who wasn't**		
participants blinded		
**11a-ii) Discuss e.g., whether participants knew which intervention was the "intervention of interest" and which one was the "comparator"**		
Wording was such that participants could not tell - both groups received dietary<br>information		
**11b) CONSORT: If relevant, description of the similarity of interventions**		
Not applicable		
**12a) CONSORT: Statistical methods used to compare groups for primary and secondary outcomes**		

Food Intake and Nutrient Analysis: Nutrient analysis was conducted using ESHA -<br>The Food Processor Nutrition Analysis and Fitness software and the Canadian<br>Nutrient File [41, 42]. Averages of the three days of nutrient values were used in the analysis. FFQ values were used to derive usual intakes of the nutrients of interest [24].<br>Descriptive and Inferential Analysis: Means (± standard deviations) or medians (and interquartile range) were reported based on a given continuous variable's<br>distribution. Subject characteristics, group comparisons, and pre/post intervention differences were analyzed using Student t-tests, binomial tests of two proportions,Fischer exact tests, and two-way repeated measures ANOVA with Bonferroni's posthoc tests where appropriate. All analyses were done on an intent-to-treat basis usingSTATA software		
**12a-i) Imputation techniques to deal with attrition / missing values**		
Outlined in previous question		
**12b) CONSORT: Methods for additional analyses, such as subgroup analyses and adjusted analyses**		
Outlined in Q 12 a)		
RESULTS		
**13a) CONSORT: For each group, the numbers of participants who were randomly assigned, received intended treatment, and were analysed for the primary outcome**		
478 persons expressed interest in study participation. Ratio of females and males that were interested in the study was ~8:1, and although all males on the recruitment list were contacted there was imbalance in the male/female participant ratio. A total of 180 (38%) were invited to complete the online eligibility screening questionnaire; 73 of the invited individuals (73/180; 41%) were deemed eligible. Of the 73 eligible individuals, 58 enrolled and 55 (95%) completed the baseline health assessment questionnaire and food records (55/73; 75%). The final sample consisted of 55 adults between 37 and 57 years old (mean=45.8 years, SD±5.8).		
**13b) CONSORT: For each group, losses and exclusions after randomisation, together with reasons**		
As detailed in previous question; also a figure provided		
**13b-i) Attrition diagram**		
Figure 2		
**14a) CONSORT: Dates defining the periods of recruitment and follow-up**		
3 months		
**14a-i) Indicate if critical "secular events" fell into the study period**		
Not applicable		
**14b) CONSORT: Why the trial ended or was stopped (early)**		
Not applicable		
**15) CONSORT: A table showing baseline demographic and clinical characteristics for each group**		
Table 1		
**15-i) Report demographics associated with digital divide issues**		
Not really applicable		
**16a) CONSORT: For each group, number of participants (denominator) included in each analysis and whether the analysis was by original assigned groups**		
**16-i) Report multiple "denominators" and provide definitions**		
Table 1 and 3		
**16-ii) Primary analysis should be intent-to-treat**		
All analyses were done on an intent-to-treat basis using STATA software [43].		
**17a) CONSORT: For each primary and secondary outcome, results for each group, and the estimated effect size and its precision (such as 95% confidence interval)**		
Table 3		

**17a-i) Presentation of process outcomes such as metrics of use and intensity of use**		
Table 3		
**17b) CONSORT: For binary outcomes, presentation of both absolute and relative effect sizes is recommended**		
Table 3		
**18) CONSORT: Results of any other analyses performed, including subgroup analyses and adjusted analyses, distinguishing pre-specified from exploratory**		
Changes in Dietary Intake, Anthropometrics, Self-E□cacy and Quality of Life<br>Signi□cant pre/post intervention differences (Table 3) were found for percent of<br>calories from total fat (mean difference {MD}=-5.1%, Wilks' lambda {□}=0.817,<br>F(1,53)=11.68. P=.001, eta-squared {□2}=0.183), as well as saturated fat<br>(MD=-1.7%, □=0.816, F(1,53)=11.71, P=.001, □2=0.18), and HRQOL scores (MD=+8.1 points, □=0.914, F(1,53)=4.92, P =.031, □2=0.086). There were<br>signi□cant differences between groups over time for sodium (□=0.846, F(1,53)=9.47, P=.003		
**18-i) Subgroup analysis of comparing only users**		
as above		
**19) CONSORT: All important harms or unintended effects in each group**		
yes but no applicable		
**19-i) Include privacy breaches, technical problems**		
Yes not applicable though		
**19-ii) Include qualitative feedback from participants or observations from staff/researchers**		
Based on insights from our qualitative data, the variable findings among intakes of vitamin and mineral intakes may be due to a lack of understanding by the participant of their functions and relevance.		
DISCUSSION		
**20) CONSORT: Trial limitations, addressing sources of potential bias, imprecision, multiplicity of analyses**		
**20-i) Typical limitations in ehealth trials**		
This study's strengths include its high retention rate, focus on a de□ned adult<br>population, assessment of dietary intakes using FFQ estimates and three-day food records to reduce misreporting error, and the provision of quantitative and<br>qualitative data. However, this investigation could have been strengthened by<br>including objective measures such as biochemical indicators of nutrient status. The modest sample size prevented strati□cation of results based on individual genes. Furthermore, given the composition of the sample were mainly female and<br>Caucasian the generalizability of the results are limited.		
**21) CONSORT: Generalisability (external validity, applicability) of the trial findings**		
**21-i) Generalizability to other populations**		
This study's strengths include its high retention rate, focus on a de□ned adult<br>population, assessment of dietary intakes using FFQ estimates and three-day food records to reduce misreporting error, and the provision of quantitative and<br>qualitative data. However, this investigation could have been strengthened by<br>including objective measures such as biochemical indicators of nutrient status. The modest sample size prevented strati□cation of results based on individual genes. Furthermore, given the composition of the sample were mainly female and Caucasian the generalizability of the results are limited.		
**21-ii) Discuss if there were elements in the RCT that would be different in a routine application setting**		
Providing participants with DNA information related to diet improved knowledge,<br>motivation, and action related to healthy eating. However, tailored practitioner-led gene-based personalized nutrition interventions tend to be more effective in<br>improving dietary intakes of key target nutrients such as fat and sodium.		
**22) CONSORT: Interpretation consistent with results, balancing benefits and harms, and considering other relevant evidence**		

**22-i) Restate study questions and summarize the answers suggested by the data, starting with primary outcomes and process outcomes (use)**		
This study compared the effectiveness of standard and tailored gene-based personalized nutrition interventions in improving knowledge and motivation to change eating behavior, dietary intakes, and quality of life. A second objective was to elicit participant feedback from focus groups in order to better understand the quantitative findings. The results indicated that over a nine-week period, tailored interventions contributed to improved fat and sodium intakes, overall diet quality, and quality of life. In addition, participants receiving these types of interventions typically indicated that they intended to make changes to their diet in the near future. When phenotypic plus genotypic information by group assignment was considered, improved total fat, saturated fat, and sodium intakes were found among those who possessed the risk genotype and received tailored dietary advice.		
**22-ii) Highlight unanswered new questions, suggest future research**		
To the best of our knowledge, the findings which showed time by group interactions for sodium and overall diet quality have not been reported elsewhere. In particular, the I-group had more pronounced reductions in dietary sodium intake compared to the C-group. These results have implications for the prevention and treatment of hypertension, a condition which can be exacerbated by high sodium intakes and progress to coronary heart disease in some individuals.<br>The findings that indicated significantly greater improvements in quality of life measures occurred in the intervention group were novel. Although, prior studies have shown relationships between poorer quality diet and worse quality of life [50], to the best of our knowledge this is the first time this has been reported related to gene-based personalized nutrition interventions.<br>It was surprising that dietary improvements were not observed across all results where nutrition-related risk variants were present in the participants. The focus group data helped to provide insights about these findings by suggesting that although participants found the information useful, it was also overwhelming. As a result, they would try to simplify the implementation of their results by adjusting intakes of the major nutrients and sodium and pay less attention to the micronutrients-based gene information. However, in doing this, they may not be substantially improving the overall quality of their diet or meeting all of their micronutrient requirements. For the future, improved nutrition education strategies are needed that will facilitate the uptake of all recommended dietary changes that are based on gene test results.		
Other information		
**23) CONSORT: Registration number and name of trial registry**		
Registration: Clinicaltrials.gov NCT03310814, http://clinicaltrials.gov/ct2/show/ NCT03310814		
**24) CONSORT: Where the full trial protocol can be accessed, if available**		
Littlejohn P, Irene M, Brown E, Afroze R, Davison KM. A comparison of nutrigenomics technology interface tools for consumers and health professionals: Protocol of a mixed methods study. JMIR Res Protoc 2018;7(6):e115. [doi: 10.2196/resprot.9846]. PMID: 29891470. Full free text available at: https://www.ncbi.nlm.nih.gov/pmc/articles/PMC6018240/		
**25) CONSORT: Sources of funding and other support (such as supply of drugs), role of funders**		
This study is funded a Natural Sciences and Engineering Research Council of Canada (NSERC) Engage Grant (Grant # 500692 – 16).		
**X26-i) Comment on ethics committee approval**		
The study protocol, including paper-based on online data collection forms, was approved by Quorum Institutional Review Board (protocol # 32220CDN/1).		
**x26-ii) Outline informed consent procedures**		
All participants provided initial online consent to collect eligibility screening information and, if eligible, baseline information related to their health. At the participant’s first site visit, a second written consent form was reviewed which participants signed to confirm their continued involvement in the study.		
**X26-iii) Safety and security procedures**		

Outlined in informed consent		
**X27-i) State the relation of the study team towards the system being evaluated**		
Not applicable		
